# A living meta-ecological study of the consequences of the COVID-19 pandemic on mental health

**DOI:** 10.1007/s00406-021-01242-2

**Published:** 2021-03-06

**Authors:** Stefan Leucht, Andrea Cipriani, Toshi A. Furukawa, Natalie Peter, Thomy Tonia, Theodoros Papakonstantinou, Alexander Holloway, Georgia Salanti

**Affiliations:** 1 Department of Psychiatry and Psychotherapy, Technical University of Munich, School of Medicine, Klinikum Rechts der Isar, Ismaningerstr 22, 81675 Munich, Germany; 2 Department of Psychiatry, University of Oxford, Oxford, UK; 3 Department of Health Promotion and Human Behavior, School of Public Health, Kyoto University Graduate School of Medicine, Kyoto, Japan; 4 Institute of Social and Preventive Medicine, University of Bern, Bern, Switzerland

The COVID-19 pandemic is a worldwide challenge for mental health [1]. On the one hand, people are afraid of being infected with the virus. On the other hand, we humans are social beings (“Zoa Politika”); therefore, containment measures leading to isolation can cause not only mental health problems such as depression, anxiety and loneliness, but also exacerbation of psychosis, drug and alcohol abuse, and domestic violence [2]. The consequence of the pandemic for mental health can be further amplified as more people are threatened by unemployment or financial insecurity. Nevertheless, some people also benefit from the positive aspects of the situation. The world’s pace has slowed down reducing distress for some people. We experience novel forms of remote communication and telework that minimize travelling and face-to-face meetings, with positive impact on the work–life balance and the environment in the long term. While for families with small apartments and young children, home office presents a major challenge, others experience an increase in quality of life due to reduced commuting or because they can spend more time with their (older) children.

**Fig. 1:**
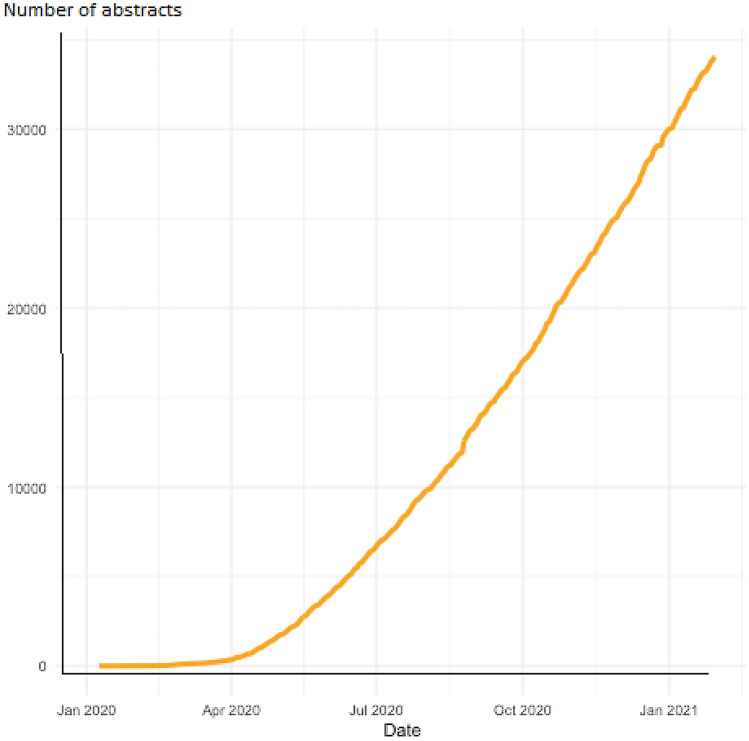
Number of abstracts about the association between COVID-19 and mental health problems (January 2020–February 2021). Details of the search strategy can be found here: https://esm-ispm-unibe-ch.github.io/covid19-mhsr/search-strategy/

Since the beginning of the pandemic, a large number of publications on the topic have accumulated and several enlightening articles are published in this special issue of the journal [3–6]. A broad search identified approximately 35,000 reports published since January 2020 (see Fig. 1). Although many of these articles are of limited relevance or quality, some of them make it into major medical journals which might not have taken place outside the times of a pandemic. Some articles even get withdrawn soon after their publication. A famous example is the retracted paper on the effects (hydroxy)chloroquine as a treatment for COVID-19 [7]. Another study appeared to show that the use of masks is useless, but was retracted later because the authors did not interpret their data correctly [8]. Differences between preprints and associated journal articles have also been reported (9). In our field, many teams have conducted cross-sectional online surveys to find out whether the pandemic led to an increase in mental health problems. However, many of these studies do not report the prevalence of the measured mental health issues before the pandemic. Without such information, it is not possible to properly assess whether the frequency of reported mental health problems have changed. Well-designed longitudinal studies which monitor these symptoms before, during and after the pandemic are needed, but they are rare.

The exact extent of the problem remains unclear, yet knowing it would be important for policy-makers to take the right decisions. It is a complicated picture which needs to take into account the prevalence of mental health problems before and during the pandemic in a specific geographical area and the population characteristics. Any excess frequency of mental health problems will also depend on the prevalence of COVID-19 cases in each area, and the level of the containment measures. One would expect that a higher risk to get infected, more severe containment measures, in particular reduction of social contact, and their resultant economic impacts (e.g. unemployment) would lead to increased prevalence of mental health problems compared to pre-pandemic levels. Moreover, there are subgroups of people with different risk of infection, based for instance on age, gender, comorbidities (e.g. people who are immunosuppressed), employment and socioeconomic status, etc., while these subgroups differ significantly in how the pandemic and restrictions interfere with their daily lives as well.

This multifaceted association warrants an approach beyond a simple meta-analysis of prevalence studies and subgrouping/meta-regression. A “meta-ecological study” that will associate changes in the prevalences of mental health problems with the intensity of the pandemic and the stringency of the containment is the only alternative to large multinational cohorts. Note that as new studies are published every day and this trend will continue as long as the pandemic endures, likely well into the foreseeable future due to many factors, including the rise of new virus variants. Therefore, the study needs to be implemented as a “living systematic review”, a review and data synthesis which is regularly updated as new studies are published. Given the enormous amount of papers that need to be screened and the data that need to be extracted in a timely manner, engaging volunteer researchers who commit some of their (free) time (“crowdsourcing”) is the most viable option to undertake the review process.

In this context, the Swiss National Science Foundation is currently funding Mental Health COVID (MHCOVID), a project in which approximately 80 “crowders” with experience in systematic reviews from all five continents (see map https://mhcovid.ispm.unibe.ch/crowd.html) are selecting and extracting data from mental health prevalence studies into an internet-based system. These data will be combined with numbers on the prevalence of mental health problems in the study area before the pandemic, with measures of the prevalence of COVID-19 infections and with measures of the stringency of the containment measures imposed on the population. Bayesian statistics will be applied to synthesize the various sources of evidence and the results will be continuously updated and presented on the project’s website (https://mhcovid.ispm.unibe.ch/). MHCOVID has developed a system which can serve as a blueprint for future meta-ecological analyses in case that another pandemic emerges. This innovative approach will provide useful insights into these complex relationships and guidance for policy-makers.

For full information on MHCOVID (and if you are interested in joining the crowd of reviewers), please visit https://mhcovid.ispm.unibe.ch/crowd.html or contact the research team via e-mail (natalie.peter@tum.de) or on Twitter: @MH_COVID.
